# Contribution of non-circadian neurons to the temporal organization of locomotor activity

**DOI:** 10.1242/bio.039628

**Published:** 2018-12-10

**Authors:** Nicolás Pírez, Sofia G. Bernabei-Cornejo, Magdalena Fernandez-Acosta, José M. Duhart, M. Fernanda Ceriani

**Affiliations:** Laboratorio de Genética del Comportamiento, Fundación Instituto Leloir and Instituto de Investigaciones Bioquímicas–Buenos Aires (IIB–BA, CONICET), 1425 Buenos Aires, Argentina

**Keywords:** *Drosophila*, sLNvs, Connectivity, Non-circadian neurons, Locomotor rhythms

## Abstract

In the fruit fly, *Drosophila melanogaster*, the daily cycle of rest and activity is a rhythmic behavior that relies on the activity of a small number of neurons. The small ventral lateral neurons (sLNvs) are considered key in the control of locomotor rhythmicity. Previous work from our laboratory has showed that these neurons undergo structural remodeling on their axonal projections on a daily basis. Such remodeling endows sLNvs with the possibility to make synaptic contacts with different partners at different times throughout the day, as has been previously described. By using different genetic tools to alter membrane excitability of the sLNv putative postsynaptic partners, we tested their functional role in the control of locomotor activity. We also used optical imaging to test the functionality of these contacts. We found that these different neuronal groups affect the consolidation of rhythmic activity, suggesting that non-circadian cells are part of the circuit that controls locomotor activity. Our results suggest that new neuronal groups, in addition to the well-characterized clock neurons, contribute to the operations of the circadian network that controls locomotor activity in *D. melanogaster*.

## Abstract (precis)

**Summary:** Here we characterized the impact of different putative postsynaptic partners of the sLNvs on the control of rhythmic locomotor behavior. We found that some of these novel neuronal clusters are relevant for the control of locomotor activity.

## INTRODUCTION

For decades, *Drosophila melanogaster* has been used as a model system to study circadian rhythms. The daily cycles of rest and activity are one of the outputs of the circadian circuit that have been used to test the functionality of the system. The evidence around the control of these cycles is ample, and in flies, the circadian network that controls this and other behaviors is relatively small, comprising around 200 neurons organized in a small number of clusters in the central nervous system (Helfrich-Förster, 2003; Kaneko and Hall, 2000). Among all the different groups, the cluster that includes the small ventral lateral neurons (sLNvs) is a key member of the circuit. These neurons are critical for the temporal organization of locomotor activity throughout the day; specifically, they are capable of directing this rhythmic behavior in the absence of any other oscillator, or even in the absence of any environmental synchronizing cues, such as light or temperature (Chung et al., 2009; Grima et al., 2004; Parisky et al., 2008; Renn et al., 1999; Shang et al., 2008; Stoleru et al., 2004, 2005). The sLNvs are believed to set the pace of other circadian oscillators in the brain, mediated in part by the release of the PDF neuropeptide (Stoleru et al., 2005). This neuropeptide and its receptor are crucial for the circadian network to function properly. Mutant flies that lack this peptide (*pdf^01^*) or the receptor to detect it (*han^5304^*) become progressively arrhythmic in the absence of external cues, display shorter locomotor activity periods and also present defects in the morphology of these neuronal projections (Gorostiza and Ceriani, 2013; Hyun et al., 2005; Im and Taghert, 2010; Renn et al., 1999). Recent experiments have shown that the PDF receptor is expressed in different neurons outside of the circadian system (Parisky et al., 2008), and that this neuropeptide is capable of activating its receptor on different structures of the brain, such as the ellipsoid body, pointing to a relevant link between the circadian and locomotor systems (Pírez et al., 2013).

It was shown that the dorsal axonal projections of the sLNvs undergo a dramatic structural remodeling on a daily basis (Fernandez et al., 2008), being far more complex during the early morning. This remodeling confers the system with an important display of plasticity. Adult-specific downregulation of different clock components in the LNvs confirmed that a functional clock is required for this remodeling to take place (Herrero et al., 2017). These projections are shorter in length and less arborized at night (Gorostiza et al., 2014). Taking advantage of the GFP reconstitution across synaptic partners (GRASP) technique (Feinberg et al., 2008; Gordon and Scott, 2009), it was shown that that the sLNv neurons contact different synaptic partners at different times along the day (Gorostiza et al., 2014), and appear to make synaptic connections with other members of the circadian network (Cavanaugh et al., 2014; Frenkel et al., 2017; Gorostiza et al., 2014; Guo et al., 2016; Tang et al., 2017). This evidence raised the question of how information about time of day is passed along to different members of the circadian network, and what is the role of the novel non-circadian cells that are being contacted by the sLNvs (Cavey et al., 2016; Gorostiza et al., 2014). As mentioned previously, the sLNvs play a key role in the timing of the morning peak, as well as in the circadian rhythms of locomotor activity (Grima et al., 2004; Renn et al., 1999; Stoleru et al., 2004). On the other hand, the large ventral lateral neurons (lLNvs) are known to be relevant in regulating the levels of arousal driven by light (Parisky et al., 2008; Shang et al., 2008; Sheeba et al., 2008).

Using a lLNvs ‘specific’ driver (i.e. C929-GAL4) Shang and colleagues showed that these neurons contribute to higher arousal levels and lower sleep in a light-dependent manner, and suggested that these neurons promote the activity of the central complex, a higher order center for locomotion (Shang et al., 2008). In support of this, the PDF receptor is expressed and active within cells of the ellipsoid body that is part of the central complex in this area (Parisky et al., 2008; Pírez et al., 2013). To study the interaction between the circadian and sleep circuits, Liu and colleagues resorted to a wide awake (wake) mutant exhibiting a marked delay in sleep onset (Liu et al., 2014). The authors suggest that the function of WAKE is to promote the initiation of sleep by means of increasing GABA sensitivity through upregulation of the GABA_A_ receptor RDL in the lLNvs during the day to night transition (Liu et al., 2014). Their data points to a relevant role of the lLNvs in the intersection between the circadian and sleep circuits, along with the locomotor system, raising the possibility of a direct communication between these cells and the main pacemaker group, the sLNvs.

Here we studied the role that putative synaptic partners of the sLNvs have on the daily rhythms of locomotor activity. Through behavioral experiments in which we altered the excitability of these cells, we show that non-clock neurons that are contacted by the sLNvs have an impact on rhythmic patterns of locomotor activity, suggesting that these neurons are part of the output pathway that executes those behaviors whose activity is coordinated by upstream clock neurons.

## RESULTS

Previous experiments from our laboratory established that the sLNvs undergo a significant structural remodeling on a daily basis (Fernandez et al., 2008). Recently, we reported that circadian pacemaker neurons make synaptic contacts with different targets throughout the day (Gorostiza et al., 2014). These results support the idea that the synaptic connectivity of pacemaker cells is under circadian control, thus implying a means to control how information about time of day is passed along the network. In this work, we analyzed putative postsynaptic partners of the sLNvs and directly tested the role these non-circadian cells play in the circuit that controls rhythmic locomotor activity.

### Constitutive silencing of non-circadian sLNv-contacting neurons triggered deconsolidation of rhythmic activity patterns

Previous work had identified a set of enhancer trap lines that contacted the sLNvs at different times in the day: *11-8*, *3-86*, *5-133*, *4-93*, *4-12* and *4-59*, that will collectively be described as GRASP+ (Gorostiza et al., 2014). Two additional lines (*7-49* and *5-43*) showed no detectable GFP reconstitution (GRASP−) and were used as negative controls (Gorostiza et al., 2014). Additionally, the GAL4 driver *OK107*, which is expressed in the α/β and γ lobes of the mushroom body (MB) and, to a lesser extent, in the *pars intercerebralis* (PI) was also used in the GRASP analysis. This line should also be consider GRASP+, since there was reconstitution in all the time points analyzed (Gorostiza et al., 2014).

Inward rectifying potassium channel (*Kir2.1*) overexpression is known to silence targeted neurons (Depetris-Chauvin et al., 2011; Nitabach et al., 2002). In order to test their functional role on the control of locomotor activity, we drove the expression of *Kir2.1* under the control of the different GRASP+ and GRASP– enhancer trap lines. *Kir2.1* expression in the *4-12*, *4-59* and *OK107* domains resulted in lethality during development, precluding the analysis of adult behavior.

**Fig. 1.**
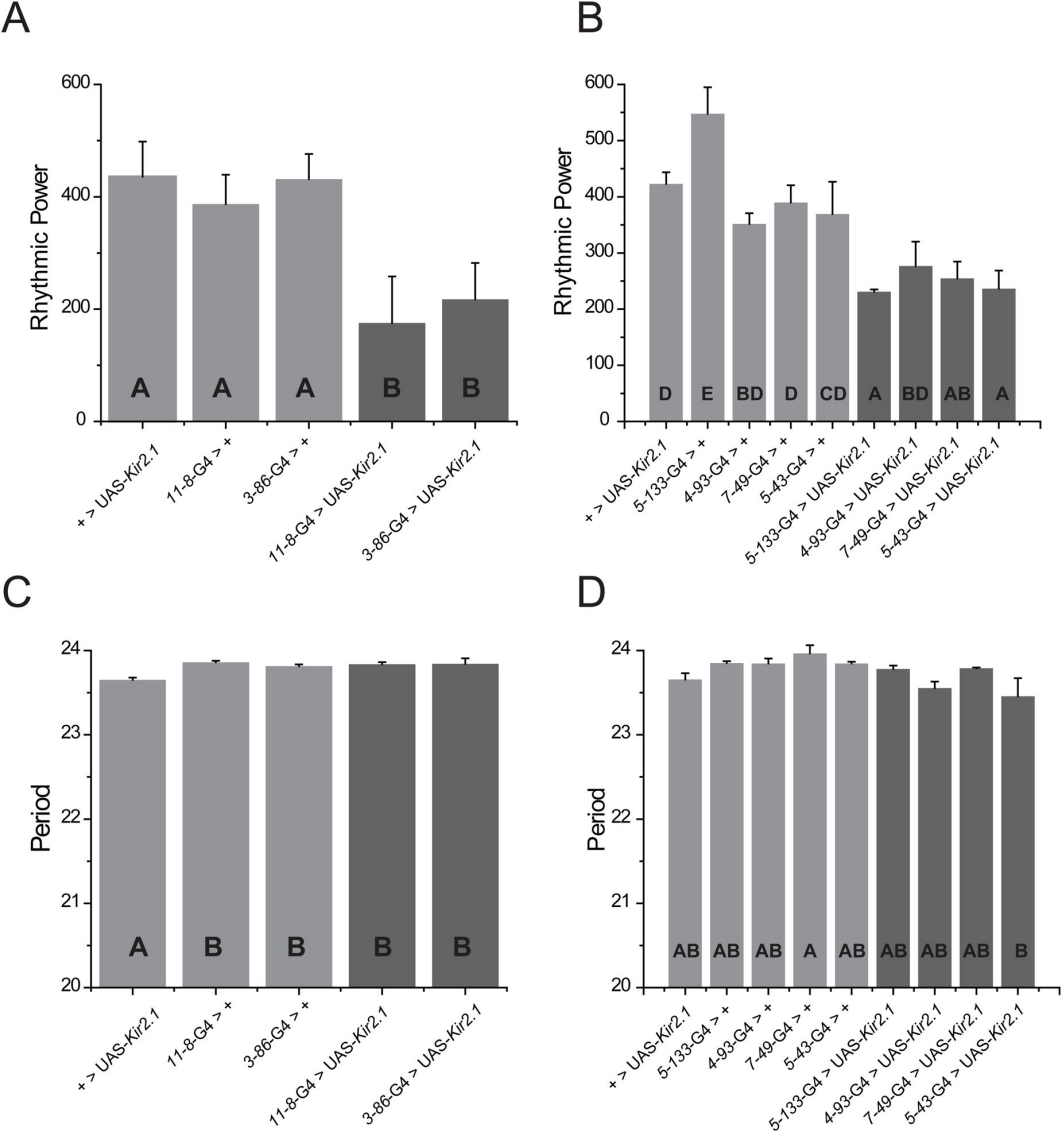
**Constitutive silencing of non-circadian neurons caused a significant reduction on rhythmic power.** Average rhythmic power and period under constant darkness (DD) for control lines (+>UAS-*Kir2.1* and *enhancer trap*-GAL4>+) and experimental lines expressing the hyperpolarizing channel *Kir2.1* under the expression pattern of the respective enhancer trap GAL4 lines. (A) Average rhythmic power for the experimental group one: *11-8* and *3-86* (one-way ANOVA, *F*_12.78127_, *P*<0.0001, genotype Tukey Comparisons, *P*<0.0001). (B) Average rhythmic power for the experimental group two: *7-49*, *5-133*, *5-43* and *4-93* (one-way ANOVA, *F*_16.75476_, *P*<0.0001, genotype Tukey Comparisons, *P*<0.05). (C) Average period for the experimental group one: *11-8* and *3-86* (one-way ANOVA, *F*_9_, *P*=0.0033, period Tukey Comparisons, *P*<0.01). (D) Average period for the experimental group two: *7-49*, *5-133*, *5-43* and *4-93* (one-way ANOVA, *F*_3.2205_, *P*=0.00187, period Tukey Comparisons, *P*<0.01). The data shown was calculated from 9–10 days at 25°C. The transition day between LD and DD was not used for these calculations. Data are expressed as mean±s.e.m. See text for a detailed explanation on the statistical analysis. Different letters represent statistical differences.

The average rhythmic power and period of flies bearing *Kir2.1* overexpression driven by *11-8*, *3-86*, *5-133*, *4-93*, *7-49* and *5-43* are shown in Fig. 1. Since the different drivers were not tested simultaneously, statistical analysis was restricted to the genotypes examined in parallel (experimental groups 1 and 2, see the Materials and Methods for a detailed explanation on the statistical analysis). Interestingly, constitutive expression of *Kir2.1* in the *11-8* and *3-86* domains resulted in a significant reduction in the rhythmic power (Fig. 1A: one-way ANOVA, *F*_12.781_, *P*<0.0001, genotype Tukey comparisons, *P*<0.0001). A similar analysis performed on the second group of drivers that included the two GRASP– lines (*7-49* and *5-43*), uncovered unexpected results (Fig. 1B). With the exception of *4-93* (a GRASP+ contact), *Kir2.1* expression in the remaining GAL4 domains caused a significant deconsolidation of the patterns of locomotor activity. The experimental lines *5-133*, *7-49* and *5-43* displayed a reduced rhythmic power compared to its corresponding GAL4 control (Fig. 1B: one-way ANOVA, *F*_16.754_, *P*<0.0001, genotype Tukey comparisons, *P*<0.05). The absence of a consistent reconstituted GFP signal between *7-49* (or *5-43*) and the sLNvs anticipated no effect on the patterns of locomotor activity (Gorostiza et al., 2014); nevertheless, these results raise new questions regarding the role of these two neuronal ensembles on the control of locomotor activity (see Discussion). An alternative explanation for these results could be that these neurons are relevant for locomotion *per se*, and the alteration of their activity is capable to alter the activity patterns, albeit not necessarily their circadian properties. On the other hand, as illustrated by *4-93*, displaying physical contacts with the sLNvs does not necessarily imply that those postsynaptic cells would play a critical role in the control of locomotor activity.

We also analyzed the contribution of the different neuronal clusters on setting the period of locomotor activity. Fig. 1C shows the period of experimental group 1 (one-way ANOVA, *F*_9_, *P*=0.0033, period Tukey comparisons, *P*<0.01). *Kir2.1* expression by both *11-8* and *3-86* drivers did not show any difference in respect to the GAL4 controls. Similar results were observed on experimental group 2; no experimental lines were significantly different to controls (Fig. 1D, one-way ANOVA, *F*_3.221_, *P*=0.00187, period Tukey comparisons, *P*<0.01), although *4-93* and *5-43* displayed a tendency towards a non-significant shorter period. To summarize, *Kir2.1*-mediated neuronal silencing elicited a clear reduction on the consolidation of rhythmic patterns in a subset of the lines analyzed *11-8*, *3-86*, *5-133*, *7-49* and *5-43*, suggesting that they might be part of a novel output circuit involved in the control of locomotor behavior.

**Fig. 2.**
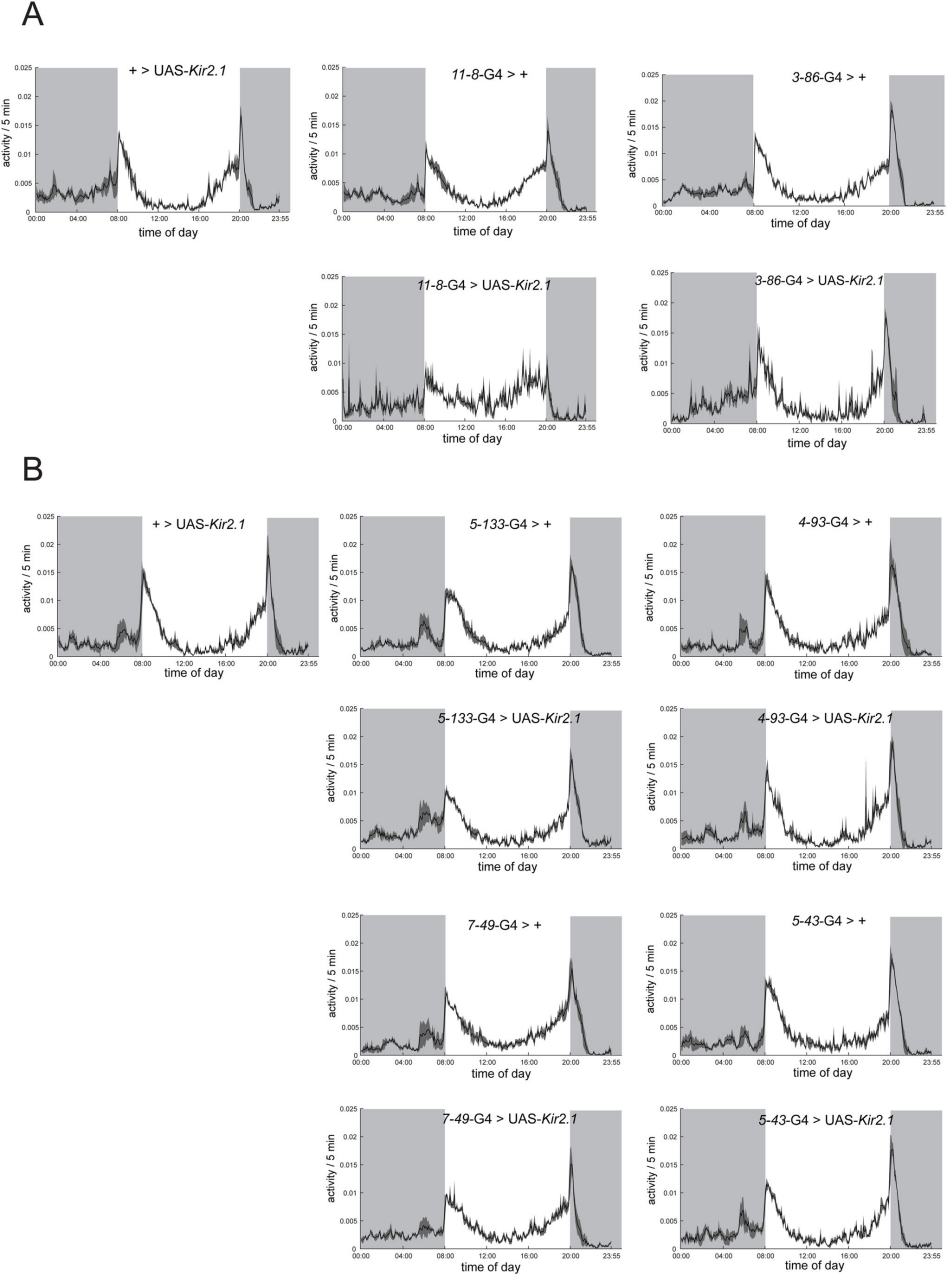
**Constitutive silencing of non-circadian neurons did not cause a significant behavioral change in the daily activity profiles.** Average activity plots for the first full day on LD for control lines (+>UAS-*Kir2.1* and *enhancer trap*-GAL4>+) and experimental lines expressing the hyperpolarizing channel *Kir2.1* under the expression pattern of the respective enhancer trap GAL4 lines. (A) Average activity plots for the experimental group one: *11-8* and *3-86*. (B) Average activity plots for the experimental group two: *7-49*, *5-133*, *5-43* and *4-93*. Shaded areas represent dark periods. See text for a detailed explanation on the statistical analysis.

To evaluate for a more subtle effect on the distribution of activity across the day, we performed average activity plots (AAPs) for the first full day on LD (Fig. 2). Visual inspection of the different AAPs shows that the experimental lines have noisier recordings, but nevertheless their activity profiles display all the features of rhythmic individuals; clear morning and evening anticipation peaks and a siesta in the middle of the day (Shaw et al., 2000; Stoleru et al., 2004). No statistical differences within control genotypes were found (experimental group 1: two-way RM ANOVA, *F*_0.9845_, *P*=0.4268, experimental group 2: two-way RM ANOVA, *F*_0.8903_, *P*=0.5043), allowing us to eliminate the UAS-*Kir2.1* from the analysis and compare each experimental line with their respective GAL4 parental control only. This analysis retrieved a similar result, no statistical differences due to genotype (experimental group 1: *11-8*: *F*_0.1749_, *P*=0.6973; *3-86*: *F*_0.7692_, *P*=0.4300; experimental group 2: *5-133*: *F*_0.7119_, *P*=0.4463; *4-93*: *F*_0.1850_, *P*=0.6893; *7-49*: *F*_0.2069_, *P*=0.6728; *5-43*: *F*_0.1118_, *P*=0.7549). In summary, this result shows that the activity profile of the animals is not affected upon overexpression of *Kir2.1*, suggesting no clear effect on the group of cells responsible for driving the morning and evening peaks.

### Acute activation of non-circadian neurons triggered deconsolidation of rhythmic activity patterns

**Fig. 3.**
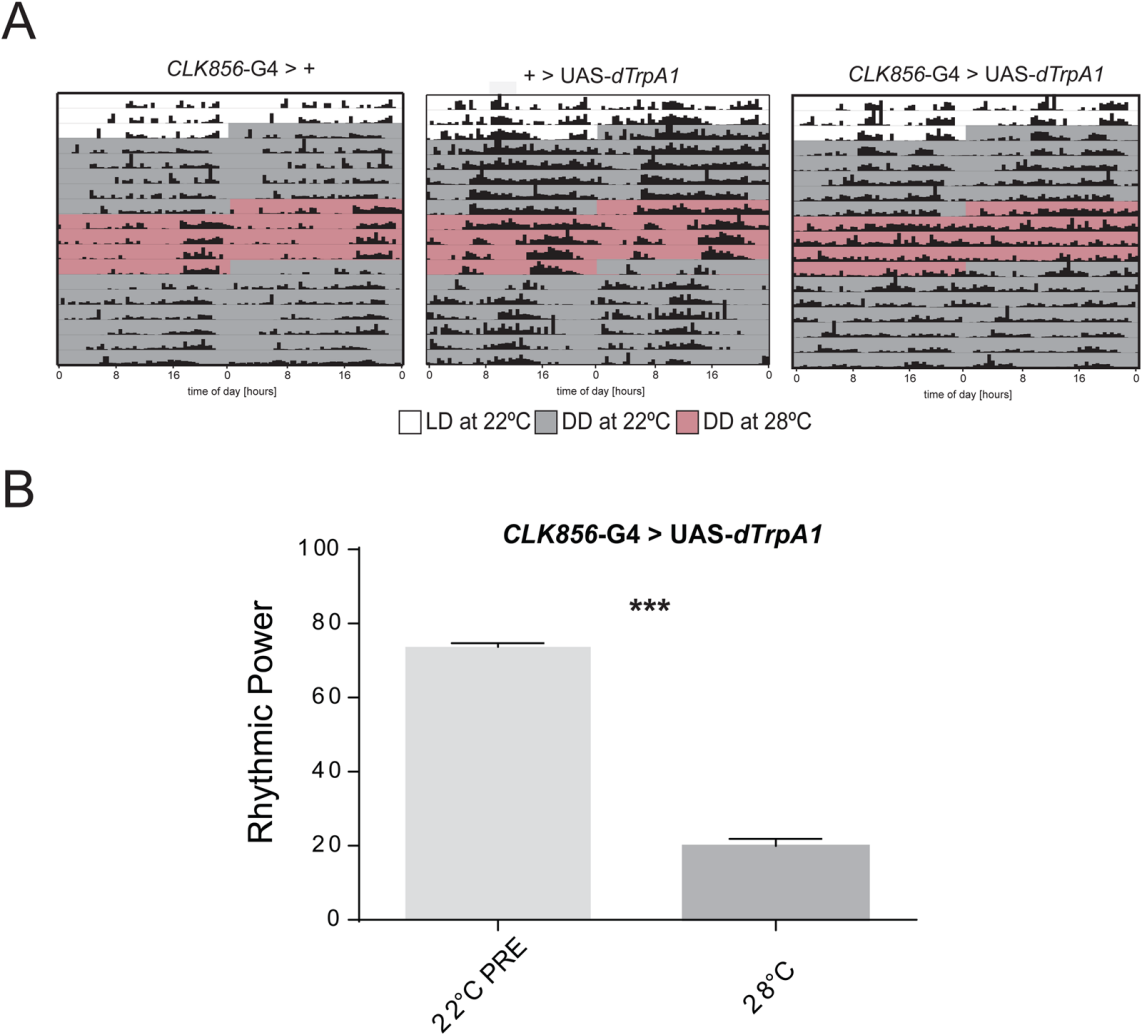
**Acute depolarization of clock neurons significantly reduced rhythmicity.** (A) Representative actograms of the indicated genotypes. The different colors represent the temperature of the experiment: 22°C (gray), 28°C (pink). (B) Average rhythmic power under constant darkness (DD) at 22°C (light gray) PRE and at 28°C (dark gray) for the experimental line expressing *dTrpA1* line under the control of the *Clk856*-GAL4. Data are expressed as mean±s.e.m. One-way ANOVA, *F*_110.3827_, *P*<0.0001, temperature Tukey comparisons, *P*<0.0001. See text for a detailed explanation on the statistical analysis.

Since chronic silencing or chronic activation could cause non-desired effects during development, the temperature-inducible tool *dTrpA1* was employed to achieve depolarization in an acute and temporally restricted manner (Rosenzweig et al., 2008). This strategy has successfully been used to identify novel circuits in the control of rhythmic behavior (Cavanaugh et al., 2014; Parisky et al., 2008; Shang et al., 2008). To test that the stimulation protocol worked properly in our hands, we expressed the *dTrpA1* channel under the control of the *Clk856*-GAL4, which restricts its expression to the central oscillators (i.e. DNs, LNd, LPN and LNvs) in the *Drosophila* brain (Gummadova et al., 2009). After the temperature was raised to 28°C, the activated *dTrpA1* channel caused a clear deconsolidation of the rhythmic pattern of locomotor activity that can be observed in the representative actograms shown in Fig. 3A. Increasing the temperature triggered some consolidation of the activity patterns at dusk in control lines. However, when rhythmic power on the experimental line was compared across the different temperatures, we observed that at 28°C was significantly lower (Fig. 3B, one-way ANOVA, *F*_110.382_, *P*<0.0001, temperature Tukey comparisons, *P*<0.0001), and partially reversible following shifting to 22°C. Nevertheless, flies expressing *dTrpA1* in the *Clk856* domain showed a clear deconsolidation of locomotor rhythmic activity. These results show that depolarization of central clock neurons results in strong and reversible behavioral changes in the pattern of locomotor activity.

**Fig. 4.**
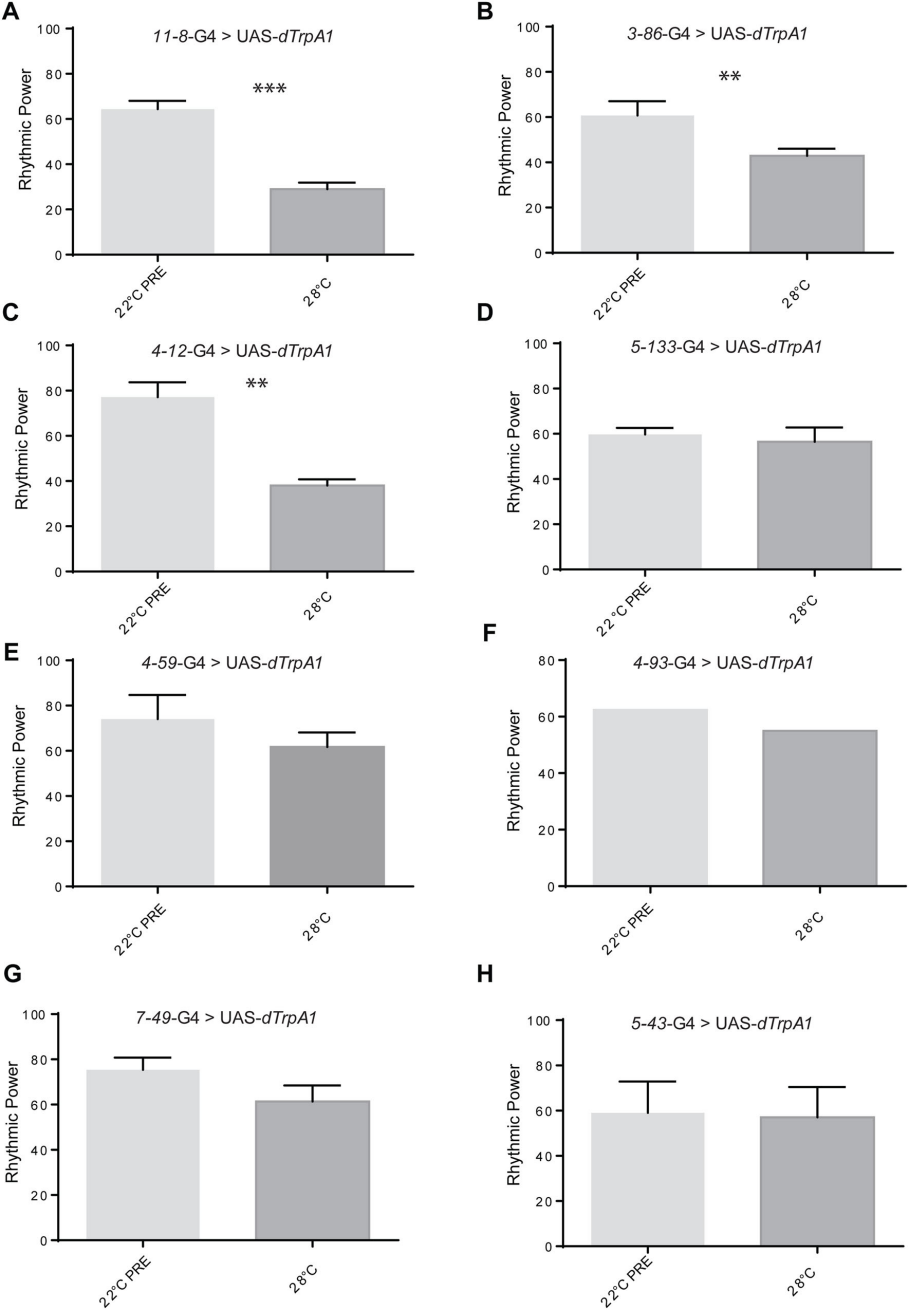
**Acute activation of non-circadian neurons triggered deconsolidation of rhythmic activity patterns.** Average rhythmic power under constant darkness (DD) at 22°C (light gray) PRE and 28°C (dark gray) for the GAL4 parental control line and experimental line, expressing *dTrpA1* line under the control of the different enhancer trap lines. (A) *11-8* (paired *t*-test, *t*_53.76_, *P*<0.0001), (B) *3-86* (paired *t*-test, *t*_5.42_, *P*=0.0123), (C) *4-12* (paired *t*-test, *t*_5.667_, *P*=0.0109), (D) *5-133* (paired *t*-test, *t*_0.6062_, *P*=0.5872), (E) *4-59* (paired *t*-test, *t*_0.9855_, *P*=0.397), (F) *4-93*, (G) *7-49* (paired *t*-test, *t*_2.619_, *P*=0.0791) and (H) *5-43* (paired *t*-test, *t*_0.2751_, *P*=0.8091). Data are expressed as mean±s.e.m. Of note, the GRASP+ *4-93* line was analyzed in a single experiment precluding any statistical analysis.

Next, we expressed *dTrpA1* under the different GRASP+ and GRASP– enhancer trap lines (Fig. 4). We analyzed this data set by means of a mixed lineal model with genotype (UAS-*dTrpA1* and *enhancer trap lines*-GAL4) and temperature as fixed factors. This initial analysis showed that there were no significant differences among the parental lines at the different temperatures (Table 1, *F*_5.1942_, *P*<0.0001); therefore, UAS-*dTrpA1* was excluded from further analysis.

**Table 1.**
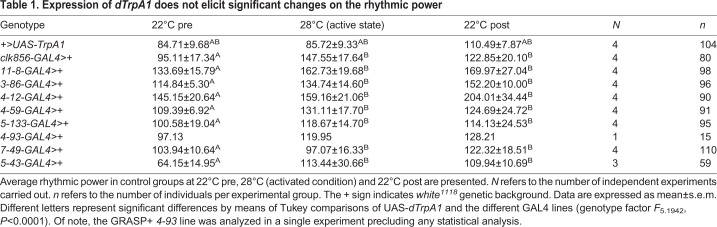
**Expression of *dTrpA1* does not elicit significant changes on the rhythmic power**

When comparing the rhythmic power at 22°C (i.e. a temperature in which *dTrpA1* is not active), versus 28°C (i.e. when is active), we found significant differences in three of the GRASP+ lines tested: *11-8* (Fig. 4A, paired *t*-test, *t*_53.76_, *P*<0.0001), *3-86* (Fig. 4B, paired *t*-test, *t*_5.42_, *P*=0.0123) and *4-12* (Fig. 4C**,** paired *t*-test, *t*_5.667_, *P*=0.0109). On the other hand, for two other GRASP+ lines differences were not significant: *5-133* (Fig. 4D, paired *t*-test, *t*_0.6062_, *P*=0.5872) and *4-59* (Fig. 4E, paired *t*-test, *t*_0.9855_, *P*=0.397), although the latter displays a non-significant reduction of the rhythmic power following the activation of *dTrpA1*.

**Fig. 5.**
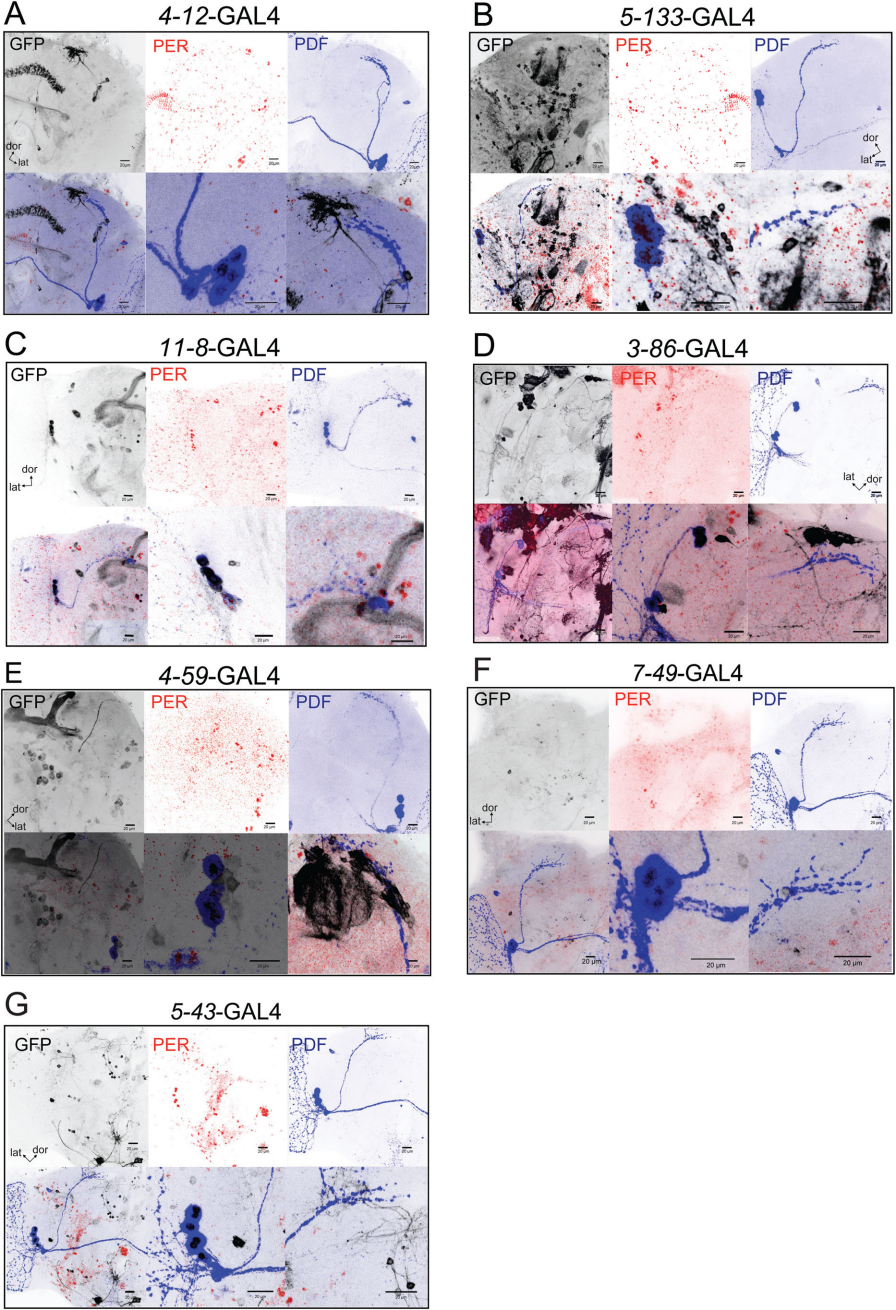
**Expression pattern of novel neuronal clusters that participate in the control of locomotor activity.** Confocal images that show a projection of the expression pattern of (A) *4-12*-GAL4>UAS-*mCD8GFP*, (B) *5-133*-GAL4>UAS-*mCD8GFP*, (C) *11-8*-GAL4>UAS-*mCD8GFP*, (D) *3-86*-GAL4>UAS-*mCD8GFP*, (E) *4-59*-GAL4>UAS-*mCD8GFP*, (F) *7-49*-GAL4>UAS-*mCD8GFP* and (G) *5-43*-GAL4>UAS-*mCD8GFP*. All figures panels are the following: upper panels from left to right: GFP channel, PER channel and PDF channel, lower panels from left to right: merge of upper panels, zoom of PDF+ somas (merge of three channels) and sLNVs dorsal projections (merge of three channels). The magnification was 40×, except upper panels of figure C that was 20×. GFP, PDF and PER signal are shown in black, blue and red, respectively. Brains were dissected at ZT=2. lat, lateral; dor, dorsal. Scale bars: 20 µm.

To assess whether activation of any given set of neurons could eventually impinge upon rhythmic locomotor behavior we evaluated two GRASP– enhancer lines, *7-49* (Fig. 4G) and *5-43* (Fig. 4H), under the same conditions. As expected, neither one of them showed any significant differences at 28°C (*7-49*: paired *t*-test, *t*_2.619_, *P*=0.0791; *5-43*: paired *t*-test, *t*_0.2751_, *P*=0.8091). In summary, these results show that acute depolarization by activation of the *dTrpA1* channel causes a clear behavioral phenotype, suggesting that non-circadian enhancer trap lines contacted by the sLNvs could be recruited in the output pathway controlling this behavior.

### Novel non-circadian clusters participate in the control of locomotor rhythmic activity in *Drosophila*

The expression pattern of the different enhancer trap lines was re-examined to confirm that no circadian neurons were included and thus could be responsible for the observed behavioral phenotypes. A membrane tethered version of GFP (*mCD8GFP*) allowed to describe the expression pattern of the different enhancer trap lines; additionally, specific markers of clock neurons were included to detect the PDF+ LNvs as well as a PER antibody as a marker of clock neurons. Fig. 5 shows representative confocal stack projections of the GRASP+ and GRASP– enhancer trap lines: A: *4-12*, B: *5-133*, C: *11-8*, D: *3-86*, E: *4-59*, F: *7-49* and G: *5-43.* The upper three panels of the figure for each genotype represent the GFP (left), PER (middle) and PDF (right) channel, showing the expression pattern across the brain of the different enhancer trap lines. The lower three panels represent the merge of upper panels (left), zoom of PDF+ somas (middle, merge of three channels) and zoom of the sLNvs dorsal projections (right, merge of three channels). For the *4-12* (*N*=4 brains) and *5-133* (*N*=4 brains) enhancer trap lines no expression of clock proteins (PER, PDF) in GFP+ cells was observed, supporting the idea that the cells that are under the control of the different enhancer trap lines are not clock neurons. This observation underscores that additional neurons, beyond the well-characterized circadian clusters, might be involved in the control of the rhythmic daily locomotor activity in *Drosophila*.

On the other hand, for *11-8*, *3-86* and *4-59* enhancer traps we observed colocalization of circadian markers in GFP+ cells (Fig. 5C–E, focus on the middle lower panel showing the PDF+ somas). This colocalization includes the sLNvs and lLNvs in the case of *11-8* (*N*=7/7 brains), whereas for the other two lines the coexpression of GFP is only localized to 2-3 lLNvs (*3-86*, *N*=5/10 brains; *4-59*, *N*=8/14 brains), in contrast to what we observed and reported earlier (Gorostiza et al., 2014). Fig. 5F and G show examples of the GRASP– enhancer traps *7-49* and *5-43*. As expected in neither of these groups GFP colocalized with either PDF or PER signals, supporting the conclusion that these two GRASP– control lines do not overlap with bonafide circadian neurons. This data shows that some of the enhancer traps support expression in a subset of the LNvs. However, this data also shows that new groups (*4-12* and *5-133*) of cells that do not include any circadian neurons contribute to the circuit that defines the rhythmic pattern of locomotor activity in *Drosophila*.

### Acute activation of the sLNvs elicited calcium responses exclusively in *OK107* neurons

**Fig. 6.**
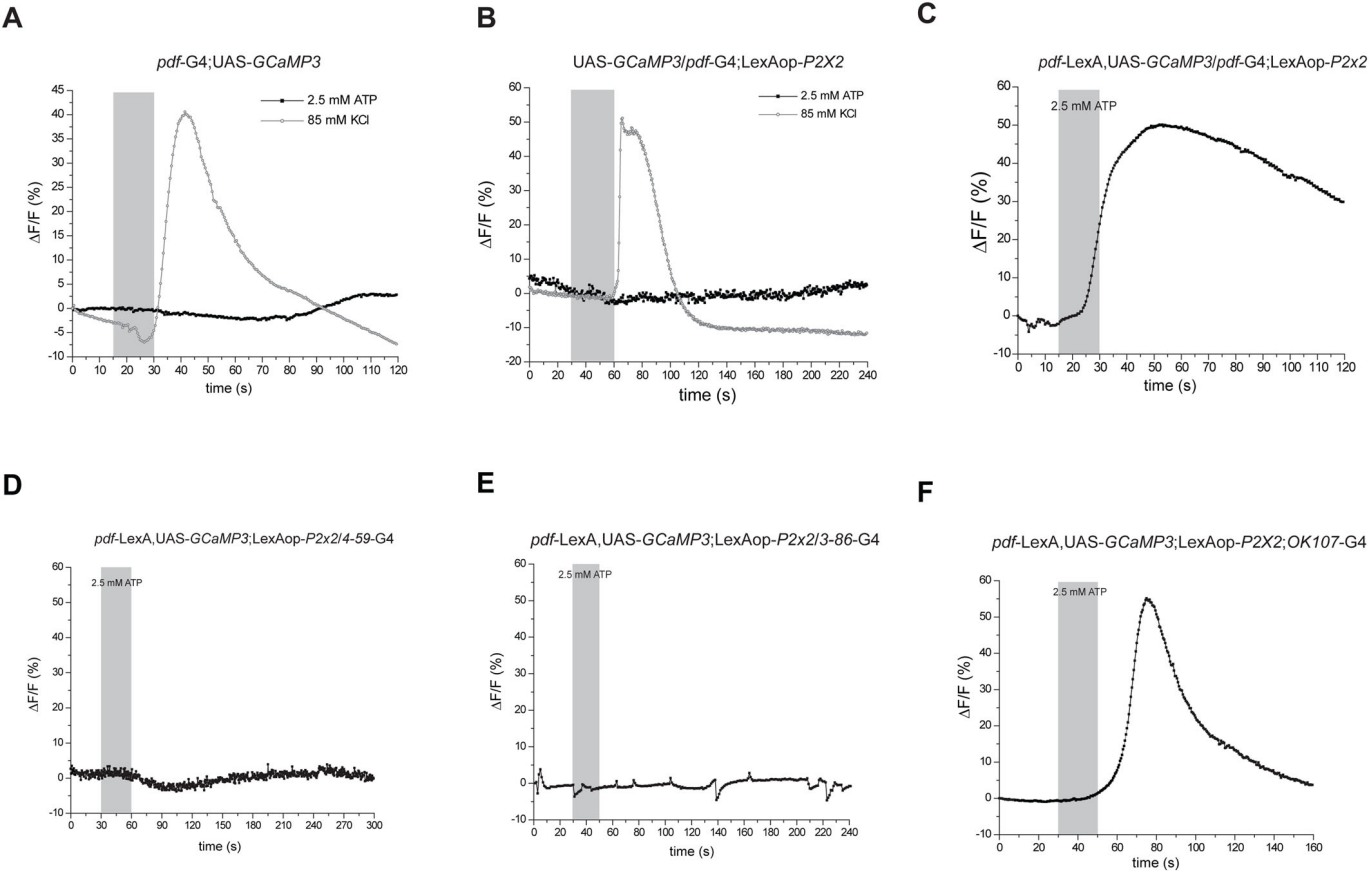
**Functionality of the synaptic contacts between sLNvs and putative postsynaptic targets.** (A) Brains that do not express the *P2X2* receptor do not show calcium changes following a stimulation with ATP (black trace). However, a high potassium stimulation does elicit a clear calcium response (gray trace). (B) Brains that express the LexAop-*P2X2* but no LexA to drive it do not show calcium changes following ATP stimulation (black trace). However, a high potassium stimulation does elicit a clear calcium response (gray trace). (C) A brief 2.5 mM ATP stimulation elicits a clear calcium response measured in the PDF+ cells. Expressing the receptor and the sensor on the same cellular group controls for the delivery system and activation of the *P2X2* receptor. *pdf*-GAL4 driver directed expression of UAS-*GCaMP3* and *pdf*-LexA that of LexAop-*P2X2*. (D–E) Perfusion of ATP (activation of the sLNvs) did not elicit significant calcium responses measured by expressing the UAS-*GCaMP3* under the control of *4-59* (D) or *3-86* (E) enhancer traps. (F) When sLNvs are activated by perfusion of 2.5 mM ATP, the mushroom body (MB) neuropil shows a clear calcium response, suggesting that the contacts between the sLNvs and the MB are functional. These experiments were performed within the ZT2–4 window. In all cases, the gray vertical bar represents the duration of the ATP stimulation.

It is possible that the structural remodeling of sLNv terminals provides the substrate for the circadian control of connectivity of PDF neurons by changing the targets they connect to and the time of the day they stay connected to them (Fernandez et al., 2008; Gorostiza et al., 2014), which would imply those contacts form functional synapses. To tackle this question we took advantage of a genetic strategy to study functional connectivity of neuronal circuits in *Drosophila* (Hu et al., 2010; Lima and Miesenbock, 2005; Schlichting et al., 2016; Tang et al., 2017; Yao et al., 2012). Here, we activated the sLNvs (i.e. ‘presynaptic’) by expressing the LexAop-*P2X2* under the control of *pdf*-LexA, while concomitantly expressing the activity reporter UAS-*GCaMP3* (Tian et al., 2009) under the control of the different enhancer trap GAL4s, to monitor changes in activity of putative postsynaptic cells in response to the activation of the sLNvs. We first performed several controls to confirm that we were able to effectively record activity following ATP perfusion and that the observed changes in fluorescence were due to the expression of the *P2X2* receptor. Specificity was assessed on brains from flies that did not express the *P2X2* receptor. As expected, no change in fluorescence was detected (Fig. 6A, black trace, *N*=5 brains). As an additional control, particularly important in non-responsive brains, after the ATP pulse brains were stimulated with a short pulse of a high potassium saline. A clear response to this stimulation (Fig. 6A, gray trace, *N*=5 brains) confirmed that the lack of response to the ATP pulse was due to the lack of the *P2X2* receptor and not due to other possibilities, such as brains not being healthy enough to respond to the stimulation. A second control was performed to test for any leaky response due to the sole expression of the *P2X2* (Fig. 6B). As expected, no signal was detected in brains that express the LexAop-*P2X2* but did not have any LexA driver. As a positive control we examined brains dissected from flies expressing both the *P2X2* channel and the Ca^2+^ reporter on the same cell population, eliciting a clear calcium increase in the cell bodies of the sLNvs detected as changes in fluorescence upon a short pulse of 2.5 mM ATP [Fig. 6C and (Yao et al., 2012)]. We recorded responses following ATP perfusion on cell bodies on 14 out 15 brains tested during the day (ZT2–ZT9) and on two out of two brains tested at night (ZT17–ZT22). The next step was to test the different enhancer traps that showed behavioral effects. Contrary to our hypothesis, ATP perfusion did not elicit significant calcium responses measured through the expression of GCaMP3 under the control of *4-59*-GAL4 (Fig. 6D, *N*=4 brains), *3-86*-GAL4 (Fig. 6E, *N*=4 brains) and *4-12*-GAL4 (data not shown, *N*=4 brains). We performed these experiments at different time points along the day to maximize the chance of success. Nevertheless, with our imaging set up and configuration we were not able to detect activation of the putative postsynaptic cells following activation of the presynaptic sLNvs.

On the other hand, when we tested the *OK107*-GAL4 we did record significant calcium responses following ATP perfusion. Fig. 6F shows an example of a successful recording from a brain that expressed the UAS-*GCaMP3* under the expression pattern of *OK107* (three out of nine brains tested). In a different set of experiments we tested the connectivity between the sLNvs and different circadian neurons [*Clk4.1*-GAL4 (*N*=3 brains), *Clk4.5*-GAL4 (*N*=5 brains), *Mai179*-GAL4>*pdf-*GAL80 (*N*=11 brains) and *tim*-GAL4>*pdf*-GAL80 (*N*=5 brains)]. Following PDF+ neuron activation, we looked for calcium responses on the somas of these different circadian neurons but we were not able to detect any significant fluorescence change (data not shown).

The activation of the MB neuropil following PDF+ neurons stimulation is shown here for the first time and allow us to confirm that the synaptic contacts between those two neuronal groups are functionally active. These results also support our hypothesis that other non-circadian neurons, such as the MB and the different enhancer traps tested here could be recruited as part of the neuronal circuit that controls locomotor behavior on *Drosophila melanogaster*.

## DISCUSSION

Rhythmic rest-activity cycles are the result of the coordinated activity of different neuronal clusters, the so-called clock neurons (Grima et al., 2004; Shafer et al., 2006; Stoleru et al., 2004; Yao and Shafer, 2014), that give rise to the specific properties of this circadian behavior (Beckwith and Ceriani, 2015; Dissel et al., 2014; Yao and Shafer, 2014; Yoshii et al., 2009). A subset of clock neurons, the sLNvs, undergo structural remodeling of its termini daily (Fernandez et al., 2008). This remodeling could represent a mechanism to change synaptic connectivity on daily basis (Gorostiza et al., 2014). Additional non-clock neurons have recently been implicated in the output pathway to rhythmic behavior (Cavanaugh et al., 2014; Cavey et al., 2016). In this work, we set out to analyze whether different neuronal clusters that are contacted by the sLNvs contribute to shape the profile of rhythmic locomotor activity of *Drosophila*. By altering neuronal excitability, we show that a small group of non-circadian neuronal clusters (i.e. *5-133* and *4-12*) does affect the locomotor activity pattern of *Drosophila*, suggesting that beyond the well-characterized clock neurons, additional, not yet characterized neuronal clusters modulate the activity of the *Drosophila* circadian network. Additionally, putative GRASP+ hits (i.e. *11-8*, *3-86* and *4-59*) include in their expression pattern circadian neurons, implying that some of the behavioral phenotypes described herein are due to deregulation of the LNvs excitability. We decided to use rhythmic power as a proxy for the rhythmicity of the population as it describes it more reliably than discrete measurements (Yao and Shafer, 2014). A significant deconsolidation of rhythmic activity and a concomitant reduction on the rhythmic power characterized several of the GRASP+ lines (*11-8*, *3-86* and *5-133*) upon *Kir2*.1 expression. Only *4-93* showed no effect upon constitutive silencing. Our results show that the line *5-133*, comprised of non-circadian neurons, contributes to the circuit controlling rhythmic locomotor behavior, presumably downstream of the sLNvs.

A surprising result was the fact that both of the GRASP– enhancer trap lines (*7-49* and *5-43*) showed a clear reduction of the rhythmic power, opening the possibility that they could play a more indirect effect on the connectivity of the circadian network (particularly in the case of *5-43* that shows a more widespread expression pattern, Fig. 5).

The fact that a neuronal group is contacted by the sLNvs does not necessarily imply that these target cells are relevant to the temporal organization of locomotor behavior, as indicated by *4-93*, suggesting that an expanded battery of behaviors should be used to uncover their function. On the other hand, affecting excitability of GRASP+ and GRASP– clusters did not result in changes in the period of individual flies, thus implying that these clusters do not mediate communication within the circadian network, a process known to alter such circadian property (Beckwith et al., 2013; Berni et al., 2008; Frenkel et al., 2017; Lear et al., 2009; Wülbeck et al., 2009). Given that some of the enhancer traps are expressed in some lLNvs, we analyzed rhythmic locomotor activity of these animals in more depth. However, average activity plots did not result in any difference between controls and *Kir2.1*-expressing flies, suggesting that the subset of lLNvs included are not contributing to shape the temporal organization of the activity. Given the relevance of the lLNvs within sleep regulation, their impact on the underlying circuit awaits further characterization. Since constitutive expression often causes compensation effects, we used the heat activated channel *dTrpA1* to depolarize neurons in a time-restricted manner. When the different GRASP+ enhancer trap lines directed *dTrpA1* expression, we observed a clear deconsolidation of rhythmic activity. As seen for the circadian neurons, this effect is reversible, although in some the recovery is only partial. On the other hand, none of the GRASP– enhancer trap lines showed significant effects upon *dTrpA1* mediated depolarization. The fact that both silencing and activation of these enhancer trap lines caused a significant behavioral phenotype supports the hypothesis that these novel non-circadian neurons are important members of the neural circuit that controls locomotor activity, probably acting as effectors of the circadian network. Surprisingly, the enhancer traps, *11-8*, *3-86* and *5-133,* triggered ‘similar’ behavioral phenotypes upon depolarization or hyperpolarization, underscoring unpredictable effects of these manipulations on the network.

Hyperpolarizing the GRASP– lines *7-49* and *5-43* had a clear effect on rhythmicity suggesting their relevance, a possibility not considered purely based on GRASP (Gorostiza et al., 2014). A simple explanation for the lack of GRASP contacts among these lines and the sLNvs would be that the connectivity among these cells is not monosynaptic. Additionally, the presence of synaptic contacts does not necessarily imply that those cells are involved in the control of locomotor activity, as exemplified by the line *4-93*. A battery of behaviors (potential outputs of the clock) should be tested to identify time of day differences that would be predicted from the direct connectivity between different ensembles of neurons.

Enhancer trap lines that affected behavior include already recognized brain areas, such as the *pars intercerebralis* (e.g. *3-86* and *11-8*) or the MB (e.g. *4-59*). The location of these structures, close to an area where multiple clock neurons, including the sLNvs and DN1s, project to, raised the possibility for direct connectivity between these integration centers (Kaneko and Hall, 2000). The PI is thought to be involved in multiple behaviors that are under circadian control but it was not until recently that a subset of PI cells were shown to be part of the circuit that controls the rhythms of activity and rest (Cavanaugh et al., 2014; King et al., 2017). As neurons from the PI are involved in the control of rhythmic locomotor activity, it is highly likely that some of the cells included in the GAL4 enhancer traps analyzed herein contribute to the phenotypes observed after the different manipulations.

On the other hand, MBs have been proposed as integration centers for multiple behaviors, which include odor recognition and learning (Dubnau et al., 2001; Keene and Waddell, 2007). It has been suggested that the MB does not contribute in dictating the rhythmicity; nevertheless, MB ablation experiments suggest that these structures could be important regulating the activity of male flies under constant darkness (Helfrich-Förster et al., 2002). By analyzing behavioral rhythmicity in flies with MB lesions (or MB mutants), Helfrich-Forster and colleagues showed that at least for entrainment and maintenance of diurnal activity rhythms, MBs are dispensable. Nevertheless, the authors suggest that the MB has an inhibitory effect on activity of male flies, but no effect on circadian activity rhythms (Helfrich-Förster et al., 2002). Thus, MBs could contribute to the control of locomotor activity and represent the anatomical substrate where the circadian, learning and memory systems interact, as suggested by the contacts between this neuropil and the sLNvs (Gorostiza et al., 2014) and our imaging experiments (Fig. 6). Thus, this connectivity would underlie the time of day modulation of learning and memory (Chouhan et al., 2015; Lyons and Roman, 2009).

Additionally, it has been suggested that blocking MB activity has a wake promoting effect by inhibiting sleep (Pitman et al., 2006). The issue is not as simple as initially thought. Recent experiments showed that within the cholinergic MB there is a subgroup of α/β core neurons that are sleep promoting and a second group of α/β surface/posterior neurons that have an opposing effect, i.e. wake promoting (Yi et al., 2013). However, the relevance of MBs remains controversial. Expressing the temperature sensitive *shibire* under the expression of MB drivers, Mabuchi and colleagues showed that blocking neurotransmission on the MB caused the flies to show arrhythmic locomotor behavior (Mabuchi et al., 2016), suggesting that MB signaling is indeed required for *Drosophila* behavioral rhythms. These results, in addition to the ‘direct’ connectivity between sLNvs and MBs (Gorostiza et al., 2014; Mabuchi et al., 2016), support our hypothesis that other neuronal clusters (i.e. enhancer trap lines tested here) could also be part of the output pathway controlling locomotor activity.

One of the goals was to test the functional connectivity of the putative synaptic contacts between the sLNvs and the different enhancer trap lines described recently (Gorostiza et al., 2014) and tested here in a behavioral paradigm. Despite this approach that enabled us to confirm the functional connectivity between the sLNvs and the mushroom body neuropil, no functional connectivity between the PDF+ cells and the different enhancer trap lines (or several circadian neurons tested) was uncovered, including the *4-59* line that supports GAL4 expression in MB neuropils. One obvious explanation points to the complexity of the MB structure, including multiple cell types that might not be in present within the *4-59* enhancer trap. On the other hand, methodological reasons could contribute to the negative outcome: changes in calcium concentration on the inside of a cell are normally associated with a depolarization of the cell membrane. This assumes that the synaptic contact between the sLNvs and the postsynaptic cells is an excitatory synapse. However, recent findings from our laboratory show that this might not be the case (Frenkel et al., 2017). The fact that these cells release glycine, an inhibitory neurotransmitter, fits perfectly with the lack of excitatory responses in putative postsynaptic neurons. Reporters that enable detection of both excitatory and inhibitory responses, such as voltage sensitive reporters should be employed instead (Cao et al., 2013; Yang et al., 2016). Another possibility raised by these negative results is that some of the contacts between the sLNvs and the postsynaptic targets detected through GRASP do not represent functional synapses. In addition, taking into account that the original screen employed split GFP tags not directed to specific subcellular compartments; it is a formal possibility that sLNvs are not presynaptic but postsynaptic on some of the pairs. New imaging experiments, activating specific enhancer traps and looking for activity on the PDF+ neurons will enable testing this possibility. Several new techniques have been recently developed that would allow us to improve this study in the future, such as the trans-tango system (Talay et al., 2017) and t-GRASP technique (Shearin et al., 2018). In conclusion, our results along with those of others (Cavanaugh et al., 2014; Cavey et al., 2016; King et al., 2017) show that additional clusters, beyond the highly characterized clock neurons, are part of the *Drosophila* circadian network controlling locomotion.

## MATERIALS AND METHODS

### Strains and fly rearing

Flies were raised in a 12 h:12 h light:dark (LD) cycle at 25°C in vials containing standard cornmeal medium. For these experiments, we use the following stocks: *w^1118^* (RRID:BDSC_5905), UAS-*Kir2.1* (Nitabach et al., 2002), UAS-*dTrpA1* (Rosenzweig et al., 2008), UAS-*mCD8GFP*, *Clk856*-GAL4 (Gummadova et al., 2009) and *OK107*-GAL4 (MB) driver) that were obtained from the Bloomington Stock Center. We used the same group of enhancer trap lines used by Gorostiza et al. (2014): *3-86-*GAL4, *11-8-*GAL4, *4-12-*GAL4, *4-93-*GAL4, *5-133-*GAL4, *4-59-*GAL4, *5-43-*GAL4 and *7-49-*GAL4. These lines were a gift from U. Heberlein (Janelia Farm, USA). For the optical imaging experiments we used the following fly lines: *pdf*-LexA (Shang et al., 2008), UAS-*GCaMP3* (Tian et al., 2009), LexAop-*P2X2*, *pdf*-GAL4 (Renn et al., 1999) (RRID:BDSC_6900), *Clk4.1*-GAL4 (Zhang et al., 2010), *Clk4.5*-GAL4, *Mai179*-GAL4>*pdf-*GAL80 and *tim*-GAL4>*pdf*-GAL80 (Emery et al., 1998) (RRID:BDSC_7126). We generated the experimental fly lines crossing the different GAL4s to the *pdf*-LexA,UAS-*GCaMP3*>LexAop-*P2X2* line. The UAS-*GCaMP3* was obtained from Janelia Farm and the LexAop-*P2X2* was a gift from O. Shafer (University of Michigan) (Yao et al., 2012). All experimental protocols were performed in accordance with relevant guidelines and ethical regulations of our institution.

### Locomotor behavior analysis

To obtain the experimental lines, males of the different enhancer trap lines were crossed to virgin females of either UAS-*Kir2.1* or UAS-*dTrpA1*. As controls, we crossed all GAL4 lines to *w^1118^* background virgin flies. Both parental lines and their progeny were kept at 25°C on a LD cycle. 1–5-day-old males were placed in small glass tubes containing standard food and monitored for locomotor activity using the DAM system (Trikinetics, USA). Flies were kept in LD conditions for 3 days for entrainment, and then shifted to constant darkness (DD) for 11 days. In principle, expression of *Kir2.1* altered the excitability of GAL4+ neurons in a chronic fashion, both during development and in adulthood. To prevent potential developmental defects or any compensation effects caused by chronic alteration of excitability we used acute activation of the temperature sensitive *dTrpA1*. For these experiments, animals were raised at 22°C on a LD cycle. At this temperature, the *dTrpA1* channel is in a closed (i.e. inactive) state. The experiment proceeded as described above, with the exception that animals were kept at 22°C during the entrainment phase and the first 5 days on DD, when temperature was increased to 28°C for 4 days. This temperature is high enough to induce the activation of *dTrpA1*. Finally, temperature was taken down again to 22°C for the last 5 days of the experiment, to test reversibility (Cavanaugh et al., 2014). In all cases, temperature was changed at CT=0, a time in which lights would have been turned ON in an LD cycle (i.e. ZT=0). As a positive control for the experimental protocol used for the *dTrpA1* experiments, we expressed this channel on the circadian network using *Clk856*-GAL4. Period and rhythmic power were estimated using ClockLab software (Actimetrics) as previously described (Beckwith and Ceriani, 2015; Depetris-Chauvin et al., 2011; Yao and Shafer, 2014). Briefly, flies with a single peak over the significance line (*P*<0.05) in χ^2^ analysis were scored as rhythmic, which was confirmed by visual inspection of the actograms; flies with more than one peak in the χ^2^ analysis were classified as weakly rhythmic and were not taken into account for calculations. Period was calculated using data collected in DD, excluding the first DD day. Data collected in the *dTrpA1* experiments were insufficient to assign a valid free running period (at least five days are required for ClockLab analysis). Rhythmic power was used as the variable to determine the rhythmicity of the population. Average activity plots (AAPs) of the *Kir2.1* experiments were calculated as follows: the data of each fly was first separated by days; the activity of each fly was normalized relative to the sum of the total activity of the day. The normalized data was averaged in order to obtain a single AAP for all the flies of a given genotype per experiment. For the plots, the AAP of different experiments was averaged and SEM was calculated.

### Dissection and immunofluorescence

Dissection and immunostaining of adult fly brains was performed at ZT2 as previously described (Depetris-Chauvin et al., 2011). The primary antibodies employed here were: (1) anti-GFP polyclonal antibody (raised in chicken, 1:500, catalog #06-896, Upstate, RRID:AB_310288), (2) anti-PER polyclonal antibody (raised in rabbit, 1:500, catalog #PER-14A, Alpha Diagnostics, RRID:AB_1875479) and (3) homemade anti-*Drosophila*-PDF (raised in rat, 1:500; Depetris-Chauvin et al., 2011). The polyclonal secondary antibodies (Jackson ImmunoResearch) were: (1) Cy2 conjugated anti-Chicken (1:250, catalog #703-225-155, RRID:AB_2340370), (2) Cy3 conjugated anti-Rat (1:250, catalog #712-165-150, RRID:AB_2340666) and (3) Cy5 conjugated anti-Rabbit (1:250, catalog #711-175-152, RRID:AB_2340607). Images were taken on either a Zeiss LSM 510 confocal or a Zeiss LSM 710 confocal microscope. After acquisition, images were processed employing LSM Image Browser (Zeiss) or Fiji, an ImageJ-based image-processing environment (Schindelin et al., 2012).

### Brain imaging and data analysis

Imaging experiments were performed using a naked brain preparation (Pírez et al., 2013; Shafer et al., 2008; Shang et al., 2011). Briefly, whole brains were dissected in ice-cold ringer, either AHL (adult hemolymph-like) or HL3 (hemolymph-like). After dissection, brains were placed on a homemade perfusion chamber and allowed to recover for a few minutes. During the whole experiment, the preparation was kept under constant perfusion. AHL ringers contained 5 mM HEPES, 4 mM NaHCO_3_, 108 mM NaCl, 5 mM KCl, 2 mM CaCl_2_, 8.2 mM MgCl_2_, 1 mM NaH_2_PO4, 5 mM Trehalose, 10 mM sucrose (pH 7.5 based on Wang et al., 2003) and HL3 ringers contained 5 mM HEPES, 10 mM NaHCO_3_, 70 mM NaCl, 5 mM KCl, 1.5 mM CaCl_2_, 20 mM MgCl_2_, 5 mM Trehalose, 115 mM sucrose (pH 7.1 based on Shafer et al., 2008). All experiments were performed using a Leica DMLFS microscope and a 63x (NA=0.9) immersion lens and the corresponding GFP excitation/emission filter set. As light source, a 470 nm LED (Tolket Argentina) was used. All the recordings were done using a CCD camera (Hamamatsu Orca C472-80-12AG) at a 2 Hz frequency with 25–50 ms exposure and 2x binning using µManager software (Edelstein et al., 2010). The change in fluorescence was calculated according to: ΔF/F=(F_n_–F_0_)/F_0_×100%, where F_n_ is the fluorescence at time point n, and F_0_ is the fluorescence at time point 0. Data was analyzed offline using custom written software in Fiji, Matlab (Mathworks) and Excel (Microsoft). Imaging was performed at different times of the day in animals entrained to a LD cycle. 2.5 mM ATP (Sigma-Aldrich) was added to the bath by a three-way valve solenoid (Cole-Palmer) that was manually controlled, or manually by using a micropipette. Baseline images were collected for 30 s before applying any drug to the brain. The experimental flies were obtained by crossing the following line w;*pdf*-LexA,UAS-*GCaMP3*;LexAop-*P2X2*/+ with the different GAL4s. The GAL4 examined were: *pdf*-GAL4, *3-86*-GAL4, *4-12*-GAL4, *11-8-*GAL4, *4-59*-GAL4, *7-49*-GAL4 and *OK107*-GAL4. Additionally we tested different circadian drivers *Clk4.1*-GAL4, *Clk4.5*-GAL4, *Mai179*-GAL4>*pdf-*GAL80 and *tim*-GAL4>*pdf*-GAL80. In all cases, we focused on the cell bodies, which are located in the dorsolateral brain, where the sLNvs project towards. In the case of *OK107*, we focused on the MB neuropil.

### Statistical analysis

Statistical analysis was performed with InfoStat (Grupo InfoStat, FCA, Universidad Nacional de Córdoba, Argentina), JMP (SAS Software), GraphPad Prism (GraphPad Software) and R (RStudio). We consider each incubator as the experimental unit; therefore, the statistical analysis was done using the mean value for each genotype in each independent experiment, with an N between 2 and 4. The *Kir2.1* experiments were performed as two different groups. Different enhancer trap lines were used on each set of experiments (experimental group 1: *11-8*-GAL4 and *3-86*-GAL4 and experimental group 2: *5-133*-GAL4, *4-93*-GAL4, *7-49*-GAL4 and *5-43*-GAL4). With this in mind, the statistical analysis of these experiments was restricted to the genotypes examined in parallel and was tested by means of one-way ANOVA or performing a mixed lineal model testing for the effect of genotype, with incubator as a random factor (RStudio, lme library). The analysis of the AAPs was performed by means of a two-way repeated measures ANOVA. Finally, the *dTrpA1* experiment was analyzed through a mixed lineal model with genotype and temperature nested as the fixed factor and incubator as a random factor (RStudio, lme library). In some cases, a paired *t*-test was used. Results are expressed as mean±s.e.m., unless otherwise indicated and different letters represent different significance groups by either Fisher, Tukey or Sidak's comparisons.
